# Indications for Acetabular and Femoral Osteotomies for the Non-Arthritic Hip

**DOI:** 10.1007/s12178-025-09996-1

**Published:** 2025-10-31

**Authors:** Patrick England, Ryan S. Selley

**Affiliations:** Division of Orthopaedic Surgery and Sports Medicine, Lavin Family Pavilion, Northwestern Medicine, 259 E Erie St 13th Fl, Chicago, IL 60611 USA

**Keywords:** Acetabular osteotomy, Femoral osteotomy, Periacetabular osteotomy (PAO), Hip arthroscopy, Hip dysplasia, Femoroacetabular impingement

## Abstract

**Purpose of Review:**

The goal of this review is to explore the role of acetabular and femoral osteotomies in the treatment of hip dysplasia and femoroacetabular impingement (FAI). We aim to answer key questions regarding the indications and outcomes associated with these procedures in managing both conditions. Hip dysplasia and FAI are often interrelated, with joint malalignment contributing to the development of pain, dysfunction, and early osteoarthritis. Acetabular and femoral osteotomies have been shown to restore proper alignment, alleviate symptoms, and delay the need for joint replacement.

**Recent Findings:**

Acetabular osteotomy is effective for improving patient symptomatology secondary to hip dysplasia by improving coverage and reducing instability. Further, it can delay or obviate the need for hip replacement. Femoral osteotomy, on the other hand, addresses excessive femoral anteversion in instability or retroversion in FAI. Both procedures are most effective when performed early in the disease process, prior to the onset of cartilage degeneration.

**Summary:**

Major takeaways include the importance of precise preoperative imaging, careful patient selection, and significant surgical expertise. Additionally, while both osteotomies can significantly improve function and delay arthritic progression, concomitant procedures like hip arthroscopy and osteochondral autograft and allograft transplantation are still being evaluated, underscoring the need for continued research in this area.

## Introduction

Acetabular dysplasia and femoroacetabular impingement syndrome (FAI) are common non-arthritic conditions associated with hip pain [1, 2]. Acetabular or developmental dysplasia of the hip (DDH), incorporates a wide spectrum of pathology characterized by a low volume acetabulum that ranges from a fixed dislocation of the hip with abnormal proximal femoral morphology to asymptomatic shallowing. FAI encompasses three primary morphologies including cam, pincer, and mixed conditions. Cam morphology or lack of femoral head-neck offset results in premature conflict with the acetabulum for a patient’s desired activities. Pincer morphology is due to global or focal anterior over-coverage of the acetabulum on the femoral head. A mixed impingement has features of both cam and pincer deformities. These irregularities in acetabular and femoral anatomy lead to abnormal contact and biomechanical forces across the hip joint leading to labral and chondral degeneration [3].

Surgical treatment for such pathologies can include acetabular and femoral osteotomies, which have been used for the treatment of a wide range of developmental and degenerative hip diseases, including DDH, Legg-Calvé-Perthes disease, and slipped capital femoral epiphysis (SCFE) [4]. The goal of acetabular and femoral osteotomies is to restore or improve hip arthrokinematics while optimizing femoral head coverage and congruency. Recent literature has demonstrated that concomitant procedures, such as hip arthroscopy, can be included in order to comprehensively address intra-articular pathology and improve outcomes [5].

The primary objective of this paper is to review recent literature regarding indications and outcomes for acetabular and femoral osteotomies and the role of concomitant procedures to address these pathologies. A thorough understanding of these nuances can help inform surgical decision making as well as advise patients on postoperative expectations.

## Evaluating the Young Adult Hip

### History

When first evaluating a patient with hip pain it is critical to obtain a thorough history and physical examination as symptoms can be vague, making it difficult to pinpoint the exact source due to complex regional anatomy and overlapping pain characteristics. The accuracy of isolated history for distinguishing between dysplasia, FAI, and other hip pathologies is limited due to the heterogeneity of hip morphology and structural complexity of the hip joint. Through a detailed history one must evaluate if there is a prior history of trauma or childhood hip disease such as DDH or SCFE. One must also gauge the extent of symptoms and their association with participation in certain activities including frequency of athletic activity and the patient’s current activity level. Prior studies have demonstrated that FAI is one of the most common causes of hip pain in athletes [6].

Snapping and locking are common mechanical symptoms associated with hip pain. In order to distinguish between hip and back pain is it also critical to evaluate for associated paresthesias or neurologic deficits. While there are overlapping symptoms between hip and back pain prior studies have demonstrated localization patterns of hip pain consisting of groin, thigh, and buttock pain in 55%, 57%, and 71% of athletes, respectively [7]. One finding indicative of intra-articular hip pathology vs. spine etiologies is the presence of groin pain with hip flexion and internal rotation activities. Patients with limited internal rotation were 14 times more likely to have hip versus spine pathology [8].

## Physical Exam

### Supine Exam

While the patient is supine, inspection allows for side-to-side evaluation to identify quadriceps atrophy, leg length discrepancy, and pelvic obliquity. Range of motion is evaluated with passive range of motion of the symptomatic hip being compared with the contralateral side. Hip forward flexion, internal and external rotation, abduction, and adduction can be evaluated with the hip extended and with the hip flexed to 90 degrees. Patients may be more likely to have FAI when there is evidence of obligate external rotation or significant limitation with internal rotation, whereas patients with hip dysplasia have increased internal and external rotation in flexion [7]. Specific maneuvers like extension/external rotation (dial test) are performed to evaluate for hyperlaxity of the hip capsule. Patients with a positive dial test were 11 times more likely to have symptomatic instability with approximately 90% of patients having evidence of labral tearing at the time of surgery [9]. Hip strength testing can also be performed in this position specifically looking at flexion, abduction and adduction.

### Prone Exam

The patient is then repositioned prone to allow for palpation of the proximal hamstring tendon, ischial tuberosity, sacroiliac joints, and lumbar spine. Internal and external rotation should be measured in a prone position with the hips extended. Hip range of motion in this position provides the examiner with a better sense for femoral torsional abnormalities. Excessive internal rotation with limited external rotation can indicate increased femoral anteversion and is associated with snapping psoas and posterior trochanteric or ischial femoral impingement [10]. Similarly, limited internal rotation and excessive external rotation can indicate femoral retroversion. The thigh foot angle can also be assessed in this position to determine if patients have any compensatory tibial torsion abnormalities that may affect foot progression.

### Lateral Exam

The patient positioned lateral allows for further evaluation of abductor tendon and trochanteric bursa. The peritrochanteric area should be palpated for tenderness and abductor strength can be assessed in this position. One can assess for hip capsule, abductor and tensor fascia lata contractures with the Modified Ober’s maneuver. Iliotibial band snapping can also be reproduced on demand by patients in the lateral position.

### Special Tests

Patients should be systematically evaluated for various maneuvers that elicit pain like FABER (flexion abduction external rotation) and FADIR (flexion adduction internal rotation). The FABER maneuver has traditionally been used to evaluate sacroiliac joint pain but has been also used to reveal anterior capsular irritation and intra-articular hip pathology. Patients with posterior pain with this maneuver are more likely to have sacroiliac issues or posterolateral greater trochanteric impingement. Recent studies have demonstrated that preoperative impingement was present in 100% of patients with FAI with anterior impingement being more common than posterior impingement [11, 12]. The FADIR exam evaluates for impingement with prior studies demonstrating an inverse relationship between the amount of passive hip internal rotation at 90° of flexion and the severity of a cam morphology. Posterior rim impingement is evaluated by extending the hip in an abducted and externally rotated position [13]. Extension/external rotation testing recreating anterior apprehension can be suggestive of hip instability while posterior pain with this maneuver can indicate ischial femoral impingement [14, 15].

### Gait and Hypermobility

Examining a patient’s gait can provide key clues to their underlying condition. Antalgic or Trendelenburg gait and abnormal foot progression should all be assessed to evaluate for potential weakness or torsional abnormalities of the femur and tibia. While nonspecific to hip pathology one can assess subtle abductor weakness or irritation which can be associated with FAI or intra-articular pathology. Hypermobility should be also assessed, using the Beighton Score, to evaluate for possible underlying connective tissue disorders or laxity leading to exacerbation of symptoms.

### Imaging

Plain radiographs, magnetic resonance imaging (MRI), and computed tomography (CT) play a critical role in the diagnosis and treatment of non-arthritic hip conditions. Standardized techniques and accurate interpretation of the above imaging modalities are crucial for effective treatment planning [16].

### Radiographs

Radiographs are the primary imaging modality for preoperative evaluation and planning. It is critical to obtain proper radiographs to evaluate for hip dysplasia versus FAI as hip dysplasia is present in up to 17% of patients that present with hip pain [17]. There are a variety of radiographic parameters to assess for hip dysplasia and FAI including femoral head coverage, orientation of the acetabulum, joint congruity, femoral head sphericity, and femoral neck shaft angle **(**Table 1**)**.

**Table 1:** Radiographic parameters used to evaluate hip dysplasia and femoroacetabular impingement

Category	Parameter	Normal	Abnormal	Description
Acetabular Coverage	Lateral Center Edge Angle (LCEA)^a^	25–33 degrees	< 25 degrees^1,2^	Angle formed from line through the center of the femoral head to the lateral edge of the acetabulum and a vertical line
	Acetabular Index (AI) ^a^	3–13 degrees	< 14 degrees	Angle formed by a horizontal line and a line through the medial and lateral edge of the acetabular roof
	Extrusion Index ^a^	17–27%	> 27%	Percentage of the femoral head width which is not covered by the acetabulum
	Anterior Center Edge Angle (ACEA) ^b^	> 25 degrees	< 20 degrees	Angle formed by a vertical line and a line through the center of the femoral head and the anterior edge of the acetabulum.
Acetabular Orientation	Posterior Wall Sign ^a^	Negative	+/- Positive	Positive if the acetabular posterior wall travels medially to the center of the femoral head
	Crossover Sign ^a^	Negative	+/- Positive	Positive if the acetabular anterior wall crosses the posterior wall
	Ischial Spine Sign ^a^	Negative	+/- Positive	Positive if the ischial spine is projected medially to the pelvic brim
Femoral Head/Neck Sphericity	Alpha angle ^c^	< 50 degrees	> 50 degrees	Angle formed by the femoral neck axis and line through the center of the femoral head and the point where the anterior head-neck contour exceeds the head radius
	Pistol grip deformity ^a^			Asphericity of the lateral femoral head-neck junction leads to typical appearance of a pistol grip
	Offset ^c^	> 10 mm	Not Described	Difference between the femoral head radius and the neck radius
	Femoral Head Neck Offset Ratio ^c^	> 0.20	Not Described	Ratio between the anterior offset to the femoral neck and the diameter of the femoral head
Joint Congruency	Shenton’s line ^a^	Intact	Interrupted	Interrupted if the caudal femoral head-neck contour and the superior boarder of the obturator foramen do not form a continuous semicircle/arc
	Lateralization of femoral head ^a^	5–15 mm	~ 16 mm	Shortest distance between the medial aspect of the femoral head and the ilioischial line
Additional Findings				
	Neck shaft angle ^a^	126–139 degrees		
	Fovea angle delta ^a^	26+/- 10 degrees		

Projection of imaging: a- AP Pelvis; b- False Profile view; c- Cross Table Axial

LCEA studies: 1 - Welton KL, Kraeutler MJ, Garabekyan T, Mei-Dan O. Radiographic Parameters of Adult Hip Dysplasia. Orthop J Sports Med. 2023 Feb 28;11(2):23,259,671,231,152,868. 2 - Kraeutler MJ, Safran MR, Scillia AJ, Ayeni OR, Garabekyan T, Mei-Dan O. A contemporary look at the evaluation and treatment of adult borderline and frank hip dysplasia. Am J Sports Med. 2020; 48(9):2314–2323

The routine preoperative work-up includes a standing anterior-posterior (AP) radiograph of the pelvis, a lateral projection of the proximal femur, such as a cross-table lateral, frog-leg view, or a Dunn view, as well as a false-profile view. The AP Pelvis is essential as one can evaluate several radiographic measurements including: lateral center edge angle (LCEA), acetabular index, acetabular wall indices, crossover sign, posterior wall sign, ischial spine sign and neck shaft angle **(**Table 1**)** [18].

The Dunn view has been considered the most appropriate primary screening view to evaluate for cam morphology with a sensitivity ranging from 71 to 96% and specificity ranging 36–90% [19]. Ganz et al. in their original PAO publication recommend obtaining an AP view of the pelvis, a false-profile view, and abduction views as the essential preoperative work up [20].

Standardized acquisition techniques of plain radiographs are of upmost importance as factors such as patient film distance, direction of the X-ray beam, and pelvic tilt and orientation can influence the interpretation of the radiographic parameters [21].

### Magnetic Resonance Imaging (MRI)

Magnetic resonance imaging (MRI) is another imaging modality that is routinely performed in the preoperative assessment of the painful hip. One adjunct that is commonly used is intra-articular gadolinium. Studies have demonstrated that MR arthrograms have higher sensitivity and specificity for the detection of intraarticular labral lesions as compared to conventional MRI [22]. However, intra-articular contrast can obscure the visualization of articular cartilage and in patients without a prior surgical history, 3Tesla MRI scans with narrow field of view images provide high quality resolution sufficient for diagnosing labral pathology. Radial sequences are beneficial in the hip as they eliminate volume averaging seen in conventional planes, this allows for circumferential inspection of the labrum, adjacent articular cartilage wear and sphericity of the femoral head-neck junction. Due to the anterosuperior location of aspherical portions of femoral head in cam morphology, there is frequent underestimation of this morphology on plain radiographs. As such axial MR images were initially used to describe the alpha angle and demonstrate the extent of cam morphology [23].

MRI is also useful to detect subtle changes of the labrum and the articular cartilage. Thorough evaluation of the labrum is critical as labral hypertrophy has been demonstrated to be a predictor for the presence of dysplasia [24, 25]. It is thought that labral hypertrophy may represent an adaptive change in response to instability around the hip and can serve as a guide for clinical decision making in hips with borderline DDH. MRI is also useful to assess the articular cartilage status. In particular, proton density sequences are preferred due to their ability to provide contrast between bone, cartilage, and joint fluid [26]. Early in the degenerative process surface fibrillation is seen more frequently than partial or full thickness cartilage defects. Thorough evaluation for cartilage degradation is critical as advanced degenerative changes have been reported as an independent risk factor for a poor outcome after PAO [27].

MRI also allows for assessment of the surrounding musculature. The iliocapsularis is considered an important dynamic hip stabilizer and frequently assessed in borderline hip dysplasia. An increased iliocapsularis-to-rectus-femoris ratio greater than 1:1 has been described as a secondary feature in DDH that can lead clinical decision making in hips with borderline hip dysplasia and a concomitant cam-type morphology to identify the predominant pathology [28].

While there is controversy regarding MRI accuracy measuring femoral torsion, an earlier study demonstrated negligible differences between MRI and CT when standardized protocol was used [29]. Familiarity with the cross-sectional imaging findings of hip dysplasia and FAI in the young adult may allow a timely diagnosis and implementation of treatment strategies, which may prevent or delay the development of early osteoarthritis.

### Computed Topography (CT) Scan

Computed topography (CT) scans are now routinely used for multiplanar 3D assessment of underlying bony morphology and surgical planning. Prior studies have demonstrated CT scans can assess acetabular parameters including center edge angles, femoral head coverage, acetabular inclination and anteversion normalized to the anterior pelvic plan [30, 31]. Further, femoral version, true neck shaft angle, and proximal head neck offset deformity can be fully characterized via CT. Currently, many surgeons utilize these values to determine the need for an isolated arthroscopy, isolated osteotomy or a combination approach. Early efforts have been made to preoperatively plan and simulate acetabular and femoral osteotomies [32, 33]. Two studies have demonstrated feasibility of CT based navigation during PAOs [34, 35]. However, these are still in early stages and differences in postsurgical and long-term outcomes have yet to be elucidated. Further research and discussion is needed regarding the role of CT scans for pre and intraoperative planning for patients with hip dysplasia and femoroacetabular impingement.

### Injections

Intra-articular hip injections have commonly been used to differentiate intraarticular hip pain from extraarticular sources based on relief of symptoms. Despite historical controversy regarding their use in diagnosing FAI [36, 37], studies have shown their effectiveness in both diagnosing and managing FAI [7]. Injections are particularly beneficial for evaluating patients with atypical symptoms and ruling out extra-articular etiologies for pain [38]. Additionally, lack of response to injections may predict poor surgical prognosis [39]. Injections have been demonstrated to provide the most relief in patients with acetabular chondral injury and the least in those with CAM impingement [39].

### Acetabular Dysplasia

Historically the diagnosis and treatment of dysplasia was based off insufficient lateral femoral head coverage based on the lateral central edge angle (LCEA) < 20 and acetabular index (AI) >10. However, as the understanding of hip dysplasia and the resultant hip instability has evolved, there was a need to better evaluate the 3-dimensional nature of the hip. The major concerns were that diagnoses based solely on LCEA could overlook anterior or posterior under-coverage. As such the Ottawa classification was developed in order to define three discrete patterns of hip instability: Anterior (A), Posterior (P), and lateral (L) [40]. The Ottawa A and P groups are classified based upon variation in acetabular version with Ottawa A hips being anteverted and Ottawa P hips being retroverted. The Ottawa L group includes patients who are considered to have dysplasia based on the traditional definition with a LCEA less than 20 degrees or LCEA 20 to 25 degrees and an elevated Acetabular Index (AI). This classification has been shown to have reasonable intra and interrater reliability [41] as well as guide surgical treatment. A recent study demonstrated that the treatment of Ottawa A hip dysplasia demonstrates similar postoperative outcomes to patients who were treated for traditional hip dysplasia with lateral under-coverage [42].

### Acetabular Osteotomy

In young active patients who have symptomatic structural abnormalities of the hip who do not respond to physical therapy, various surgical interventions have been developed to reduce pain, improve function and minimize the risk of secondary degenerative changes [43–52]. There has been an evolution of various acetabular or pelvic osteotomies that have been utilized. Those developed to correct acetabular dysplasia include the Salter osteotomy [43], Triple innominate osteotomy [44–46], rotational acetabular osteotomies [44, 47, 48], and the Shelf Osteotomy [49–51]. The above named osteotomies are most frequently employed for treating dysplasia in skeletally immature patients. The Bernese periacetabular osteotomy (PAO), as first described by Ganz et al., has become the preferred osteotomy to address hip dysplasia in the skeletally mature patient. This powerful reorientation procedure allows for deformity correction in all planes, preservation of the pelvic ring through an intact posterior column, maintenance of acetabular blood supply and hip abductor musculature attachments [20, 44, 52]. This operation has also become the primary acetabular realignment osteotomy because of its ability to permit less restrictive postoperative weight-bearing restrictions.

### Indications

The primary indication for PAO is a patient who has had symptomatic hip pain greater than 6 months refractory to physical therapy, radiographic evidence of spherically congruous acetabular dysplasia, evidence of minimal to no osteoarthritis, and closed triradiate cartilages [44, 53, 54]. Historically the extent of dysplasia was evaluated by preoperative maximum LCEA and roof arc angle, which usually ranged from 10° to 30° and greater than 15 degrees, respectively [53, 54]. Patients may have evidence of mixed morphology, such as femoral asphericity due to CAM morphology, which can further accelerate the degenerative process.

### Rehabilitation

Postoperative rehabilitation after PAO is critical to prevent prolonged impairments in hip strength. Understanding rehabilitation parameters, such as range of motion precautions, exercise progression, and objective metrics for clearance to return-to-sport, is crucial to maximize postoperative recovery. However, there is a paucity of literature regarding appropriate progression. A recent Delphi study demonstrated that there was consensus on protected weightbearing with 25% or flat-floot weight bearing for 6–8 weeks [55]. After which time patients may wean off assist devices as tolerated if there are no gait deviations such as antalgic gait, Trendelenburg gait, or abductor lurch. There was also agreement that patients should have range of motion restrictions and be limited to 90 degrees of hip flexion and 20 degrees of external rotation for 4–6 weeks. Patients may begin upright stationary biking 6–8 weeks post-operatively and begin using an elliptical by 10–12 weeks. Before returning to sport patients must have met objective guidelines including: uninvolved hip abductor strength ratio of >80%; performance on functional tasks (i.e. single leg squat, Y-balance); and performance on sport specific drills chosen based on patient specific demands [55]. Standardized rehabilitative care following PAO is essential for achieving optimal patient reported outcomes.

## Postoperative Assessment

### Patient Reported Outcomes

PAOs have demonstrated reliability and durability in treating patients with hip dysplasia at short, mid, and long-term follow-up [56–58]. Ziran et al. reported that of the 302 hips in their study, 86% and 60% were able to avoid THA at 10- and 20-years, respectively [58]. Several factors were associated with an unfavorable outcome, greater age, lower preoperative Merle d’Aubigné-Postel score, the presence of a Trendelenburg sign, aspherical head, OA, subluxation, postoperative acetabular retroversion, excessive acetabular anteversion, and undercoverage [56]. Historically PAO study outcomes have focused on conversion to THA, however recent studies have evaluated patient reported outcomes including improvement in muscle related pain, and changes in physical function. Jacobsen et al. demonstrate that within the first year after a PAO there is a large decrease in prevalence of hip/groin related pain (74% preoperatively versus 35% 1 year after PAO) [59]. Recent prospective studies mirrored these results demonstrating that clinical success after PAOs was achieved in 95% of patients with patients reporting significant improvements in the modified Harris hip score and patient acceptable symptom state review [60]. Clinical failure was more common among patients that had undergone a prior failed arthroscopy.

### Radiographic Correction

While conversion to THA and patient reported outcomes are important in the postoperative evaluation of PAOs, it is critical that surgeons also assess restoration of the acetabulum to normal or near normal radiographic parameters. Prior studies have demonstrated that over 78% of PAOs improve radiographic parameters to be within the normal range as assessed by LCEA, ACEA, AI, and EI. For hips with an inadequate correction, the LCEA was more often undercorrected, whereas the ACEA was more often overcorrected [61]. Other studies have demonstrated that postoperative joint space width of 3 mm or less, and postoperative center-edge angle of less than 30° or more than 40° predicted conversion to THA [62]. Similarly, later studies demonstrated good patient reported outcomes when proper postoperative LCEA and Tönnis angles were obtained (LCEA < 38°, Tönnis angle of − 10° to 0°) [44, 58, 63].

### Range of Motion

Proximal femoral pathomorphologies often present in hips with DDH and FAI and a thorough understanding of this is needed as these variations can result in painful restricted ROM after PAO. The majority of patients with DDH and FAI had a positive hip impingement test prior to PAO, and initially after PAO, however this decreased to less than 20% after 5-years postop [64]. However, one must be aware that while a PAO reduces the typically increased flexion, internal rotation, and abduction in dysplastic hips, the procedure can increase the prevalence of secondary impingement of an aspherical femoral head and extraarticular impingement of the anteroinferior iliac spines in flexion and internal rotation. Patients with cam-type morphology can develop anterior impingement with restriction of internal rotation postoperatively [65]. The goal following PAO is to maintain or improve the patients pre-operative range of motion, typically this means 90–95 degrees of flexion, and 20–30 degrees of internal rotation. This often requires trimming of the acetabular fragment, subspine decompression and proximal femoral osteochondroplasty.

### Femoral Osteotomy

Femoral osteotomies are commonly employed in the setting of hip dysplasia or FAI when there is a focus to address dysplastic femoral anatomy which can lead to altered hip biomechanics. Femoral head-neck offset deformity can be treated with minimally-invasive measures such as with hip arthroscopy which will be discussed in later sections. However, femoral derotational osteotomies (FDO) are commonly used in patients who have failed hip arthroscopy or in the setting of significantly altered femoral version and foot progression alterations.

Femoral version is the angle between the femoral neck axis and the distal femoral condyles. In skeletally mature patients, normal femoral anteversion ranges from 10 to 20 degrees, with relative retroversion to be defined as less than 5 degrees of anteversion and true retroversion less than 0 degrees [18]. Patients with femoral version outside of normal parameters may have less successful outcomes after hip arthroscopy compared to patients with normal femoral version [66]. Recent studies have demonstrated improvement in patient-reported outcomes with FDO for symptomatic FAI in the setting of femoral retroversion [67]. While there are numerous types of osteotomies of the femur that have been developed this review will focus on proximal femoral osteotomies with either an intertrochanteric or subtrochanteric osteotomy.

### Indications

Proximal femoral derotational osteotomies are indicated for patients with symptomatic excessive version of the femur. Prior studies have concluded that treatment of femoral version should be based on the symptomatology, and if patients are asymptomatic, no prophylactic surgery is necessary [68]. However, the most common indications for these osteotomies has been impingement and in the setting of hip dysplasia for which many surgeons have begun to focus on reorientation of the acetabulum [11].

There are varied indications for performing femoral osteotomies both in terms of location and implant choice depending on the goals of correction and surgeon preference. The ideal correction when performing femoral sided work is not clearly defined in the literature. We typically only correct a femoral torsional deformity if either in-toeing or out-toing is present. Further, correcting to a “normal” value of 10 or 20 degrees of total femoral version would often be too severe of a correction. We generally attempt to neutralize both the foot progression angle and rotational profile of the hip with the goal of achieving a stable, impingement free hip.

Intertrochanteric osteotomies require a blade plate or proximal femoral locking plate for fixation. The author’s preference is for blade plate fixation due to implant strength and ability to compress the osteotomy site to minimize non-union risk. Indications for intertrochanteric osteotomy include the need to correct coxa valga or vara and/or need for rotational correction dependent on the type of deformity. When a purely rotational correction is necessary the level of osteotomy is selected with consideration of tension on the iliopsoas. For example, in a highly anteverted femur the iliopsoas is often overly tensioned and can be a pain generator, a retroverting intertrochanteric osteotomy allows for decompression. Conversely, in a retroverted femur the authors prefer an anteverting subtrochanteric osteotomy to avoid iatrogenic over tension of the psoas. Nonetheless, anteverted femurs can be corrected with a subtrochanteric osteotomy, the appropriate level for osteotomy selection is an area of active study.

Subtrochanteric osteotomies are best for isolated rotational deformities in the axial plane distal to the lesser trochanter. They can be secured with a standard 4.5 plate, blade plate or intramedullary nail. Plate or nail fixation each have risks and benefits. The benefit of standard plate fixation includes less lateral prominence and trochanteric irritation than with a blade plate, further it is technically less demanding. When compared to an intramedullary nail the main disadvantage of plating is the need to dissect the vastus lateralis and potentially disrupt the periosteal blood supply. Intramedullary nail fixation has the advantage of being low profile, with smaller incisions and immediate weight bearing if not combined with a Bernese periacetabular osteotomy. This comes at the cost of abductor violation and irritation from interlocking screws. Lastly, when comparing intertrochanteric and subtrochanteric osteotomies, subtrochanteric osteotomies generally tend to heal slower as they are located in cortical bone as opposed to cancellous in the intertrochanteric region.

### Rehabilitation

Similar to acetabular osteotomies understanding postoperative rehabilitation parameters is crucial to maximize postoperative recovery. Most post-operative protocols are dictated by individual surgeon preference and implant choice. Subtrochanteric derotational osteotomies performed over an intramedullary nail can weight bear as tolerated immediately while intertrochanteric osteotomies secured with a proximal femoral locking or blade plate typically require 6 weeks partial weight bearing followed by progressive weight bearing as tolerated. Concomitant procedures may also dictate periods of limited weight bearing. Prior studies have reported their rehabilitation protocols include mobilization by postoperative day 2, partial weight bearing on the operative side for 6 weeks followed by full weight-bearing with one crutch until clinical and radiographical full union [69]. Improvement in implant design has allowed for earlier weightbearing with later studies reporting immediate full weight bearing with crutches with a minimal risk of implant failure [70]. There have been more recent approaches to standardize rehabilitation in patients undergoing hip preserving surgeries starting with protected weightbearing for multiple weeks with gradual progressive hip range of motion followed by full weightbearing [71].

## Postoperative Assessment

### Patient Reported Outcomes

Multiple studies have demonstrated successful outcomes for symptomatic patients after femoral derotation osteotomy. There have been several retrospective case series of patients undergoing femoral osteotomies reported in the literature with promising results [70, 72–74]. Buly et al. report a series of 55 derotational subtrochanteric osteotomies with 75% achieving an excellent result, 23% good and 2% fair. They did however report 3 failures and a relatively high reoperation rate of 78% mainly for removal of hardware [72]. Mastel et al. reported that patients with decreased femoral anteversion, a subset of patients that are at high risk of poor outcomes after hip arthroscopy, have clinically improved pain and hip scores at 1 year post surgery [67, 75]. Similar results were seen in patients with high anteversion who underwent a subtrochanteric FDO with minimum two year follow up [12]. While heterogenous and limited by the small sample sizes, a recent meta-analysis demonstrated consistent postoperative improvements after FDOs with various hip outcome scoring systems including Subjective Hip Value, MdA score, and iHOT-33 [11]. The most commonly reported postoperative complication was hardware removal which may occur in up to 68% of patients. FDO is a reliable surgery in the definitive management of FAI with improvement in patient reported outcomes, however the limited and heterogenous sample sizes highlight the need for larger prospective studies.

### Range of Motion

There is a considerable variation in patient specific version and hip range of motion but there have been consistent improvements in hip range of motion postoperatively after FDO. Patients with retroverted femurs were noted to have approximately 23 degrees of correction in internal rotation, while as those with anteverted femurs were noted to have a 15 degree correction. Similarly, patients with retroverted and anteverted femurs were noted to have corrections in external rotation of 23 and 5 degrees, respectively [11]. These alterations are associated with a significant decrease in hip impingement, ranging from 69 to 97%. Lerch et al. demonstrated that there was an 88% reduction in patients with positive FABER testing postoperatively [12]. These changes in ROM have clinical significance such as less in-toeing and improved gait patterns.

## Concomitant Procedures

### Arthroscopy for Dysplastic Patients

Despite the success of isolated PAO for the treatment of hip dysplasia, some question the necessity of treating the high frequency of intra-articular pathology often accompanying hip dysplasia. Multiple studies have demonstrated a large portion of patients with dysplastic hips have a labral tear [76, 77]. Hip arthroscopy is a procedure that can be used to address intra-articular pathology in conjunction with PAO. Recent students have demonstrated promising results for athletes and nonathletes alike [5, 78]. However, controversy remains as a recent randomized controlled trial failed to show any significant clinical benefit in performing hip arthroscopy at the time of the PAO at 1 year follow-up [79].

Some authors have suggested that borderline dysplastic hips with concurrent labral pathology should be treated arthroscopically. However, there are limitations to hip arthroscopy, as arthroscopy alone many times is not sufficient to address the underlying pathology. As such PAO has demonstrated improved patient reported outcomes after failed hip arthroscopy. Patients that failed hip arthroscopy alone frequently demonstrated moderate-to-severe dysplasia with LCEA < 20° and minimal osteoarthritis (Tönnis grade 0 or 1) [80]. In addition, the level of preoperative pain was not associated with labral pathology, suggesting that labral pathology may not alone explain the dysplastic pain complex [81].

### Arthroscopy for FAI

Hip arthroscopy is the most commonly employed surgical treatment for FAI, unless there are patient specific contraindications. A recent meta-analysis demonstrated that patients who undergo hip arthroscopy have improved pain, function, radiographic parameters, with high rates of return to sport after surgery [11, 82]. Mastel et al. demonstrated significant improvement in patient-reported outcomes and clinical findings with either FDO or hip arthroscopy for FAI in the setting of decreased femoral anteversion as such more data is needed to determine patient-specific approaches to optimize surgical protocol and outcomes in this challenging population [75].

### Surgical Hip Dislocation

Surgical hip dislocation was the first comprehensive surgical intervention available for addressing bony impingement and intra-articular labral pathology, however this has become less frequently utilized as arthroscopy gained popularity. This procedure includes resection of the cam morphology at the femoral head-neck junction, and, if necessary, acetabular rim trimming with reattachment of the labrum. Steppacher et al. demonstrated reliable mid-term outcomes with 91% of patients showing no progression of osteoarthritis or conversion to total hip arthroplasty at 5 years [83]. Indications have now narrowed to include complex intra-articular and extra-articular bony morphologies, cartilage transplantation and revision hip preservation. In spite of declining popularity, Trotzky et al. demonstrated that surgical hip dislocation still provides consistent outcomes in the primary and revision setting with 97% of patients free from conversion to total hip arthroplasty at 5 years [84].

### Cartilage (OATS, OCA)

Osteochondral autologous transfer (OAT) and osteochondral allograft (OCA) transplantation are additional procedures that have been more recently evaluated in the treatment of patients with cartilage damage in the hip. Multiple case series have reported successful short-term outcomes in patients with symptomatic cam impingement and extensive cartilage damage (lesions >2 cm^2^) of the femoral head who were treated successfully with OAT and femoral neck osteochondroplasty [85]. A systematic review demonstrated that there is variable survivorship, ranging from 61 to 96%, in patients with less than 5 years of follow up [86].

OCA transplantation in the knee has been extensively studied and is associated with long-term success, but there is a paucity of literature of OCA for the hip. The present literature, which primarily consists of case series, suggests that this procedure may be most beneficial for patients less than 30 years old with focal cartilage defects (>2.5cm^2^) and without evidence of more global osteoarthritis. Case series with short term follow up have demonstrated variable results with prevention of conversion to THA ranging from 70 to 87.5%, however further long term follow up and larger studies are required [86, 87].

## Conclusions

Acetabular and femoral osteotomies are powerful corrective procedures for the surgical management of non-arthritic hip pain in patients with notable bony deformity. Concomitant procedures like hip arthroscopy, OATS and OCA, can be used to supplement the above procedures in addressing acetabular and femoral dysplasia, however further research is required to refine their added value. A thorough understanding of the principles of pelvic and femoral osteotomies is essential to choose the correct intervention for each patient.

## Key References

- Ziran N, Varcadipane J, Kadri O, et al. Ten- and 20-year survivorship of the hip after periacetabular osteotomy for acetabular dysplasia. J Am Acad Orthop Surg. 2019;27(7):247–255. doi:10.5435/JAAOS-D-17-00810.
  - This study demonstrated long term follow up data for PAO with good survivorship at 20 years.
- Beaulé PE, Verhaegen JCF, Clohisy JC, Zaltz I, Stover MD, Belzile EL, Sink EL, Carsen S, Nepple JJ, Smit KM, Wilkin GP, Poitras S. The Otto Aufranc Award: Does Hip Arthroscopy at the Time of Periacetabular Osteotomy Improve the Clinical Outcome for the Treatment of Hip Dysplasia? A Multicenter Randomized Clinical Trial. J Arthroplasty. 2024 Sep;39(9S1):S9-S16.
  - This study was a recent randomized controlled trial that challenged the benefit of hip arthroscopy as an adjunct for PAO treatment of symptomatic hip dysplasia without evidence of clinical benefit at 1-year follow-up. However longer follow-up will be required to determine if hip arthroscopy provides added value.
- Nelson CT, Reiter CR, Harris M, Edge C, Satalich J, O’Neill C, Cyrus J, Vap A. Femoral rotational osteotomy for femoroacetabular impingement: A systematic review. J Orthop. 2024 Dec 22;50:139–148.
  - This systematic review aggregated outcomes of several studies evaluating femoral rotational osteotomies (FDO) for FAI and demonstrated and demonstrated that FDO treated FAI successfully however has a significant amount of hardware removal (up to 40%).

## Data Availability

No datasets were generated or analysed during the current study.
